# Towards standardizing retinal optical coherence tomography angiography: a review

**DOI:** 10.1038/s41377-022-00740-9

**Published:** 2022-03-18

**Authors:** Danuta M. Sampson, Adam M. Dubis, Fred K. Chen, Robert J. Zawadzki, David D. Sampson

**Affiliations:** 1 Surrey Biophotonics, Centre for Vision, Speech and Signal Processing and School of Biosciences and Medicine, The University of Surrey, Guildford, GU2 7XH UK; 2 NIHR Biomedical Research Centre at Moorfields Eye Hospital NHS Trust and UCL Institute of Ophthalmology, London, EC1V 2PD UK; 3 Centre for Ophthalmology and Visual Science (incorporating Lions Eye Institute), The University of Western Australia, Nedlands, Western Australia 6009 Australia; 4 Department of Ophthalmology, Royal Perth Hospital, Perth, Western Australia 6000 Australia; 5 Ophthalmology, Department of Surgery, University of Melbourne, Melbourne, Victoria 3002 Australia; 6 Department of Ophthalmology & Vision Science, University of California Davis, Sacramento, CA 95817 USA; 7 Surrey Biophotonics, Advanced Technology Institute, School of Physics and School of Biosciences and Medicine, University of Surrey, Guildford, Surrey GU2 7XH UK

**Keywords:** Imaging and sensing, Biophotonics

## Abstract

The visualization and assessment of retinal microvasculature are important in the study, diagnosis, monitoring, and guidance of treatment of ocular and systemic diseases. With the introduction of optical coherence tomography angiography (OCTA), it has become possible to visualize the retinal microvasculature volumetrically and without a contrast agent. Many lab-based and commercial clinical instruments, imaging protocols and data analysis methods and metrics, have been applied, often inconsistently, resulting in a confusing picture that represents a major barrier to progress in applying OCTA to reduce the burden of disease. Open data and software sharing, and cross-comparison and pooling of data from different studies are rare. These inabilities have impeded building the large databases of annotated OCTA images of healthy and diseased retinas that are necessary to study and define characteristics of specific conditions. This paper addresses the steps needed to standardize OCTA imaging of the human retina to address these limitations. Through review of the OCTA literature, we identify issues and inconsistencies and propose minimum standards for imaging protocols, data analysis methods, metrics, reporting of findings, and clinical practice and, where this is not possible, we identify areas that require further investigation. We hope that this paper will encourage the unification of imaging protocols in OCTA, promote transparency in the process of data collection, analysis, and reporting, and facilitate increasing the impact of OCTA on retinal healthcare delivery and life science investigations.

## Abstract (web summary)

Standardized imaging protocols, data analysis methods, reporting of metrics and trial outcome measures will enhance the impact of OCTA on improving retinal healthcare.

## Graphical abstract

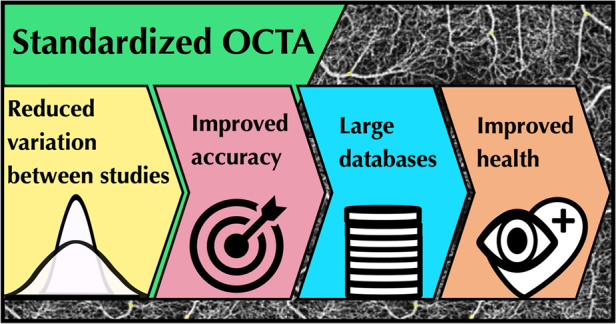

## Introduction

Biomedical optics offers many promising tools for clinical medical imaging and diagnostics that are attractive because they are non-invasive, portable, and often low cost^1^. New advances in optics and photonics technologies will continue to shape the future of biomedical research and clinical applications^2^. To fully capitalize on the potential of these promising tools to contribute to better human health, much more effort is needed in introducing and applying standards: in instrumentation, imaging protocols, data analysis methods, and reporting of findings. Such standards would facilitate open data and software sharing, and cross-comparison and pooling of data from different studies^3–6^, which, in turn, would facilitate the building of large, annotated image databases derived from healthy and diseased subjects. The availability of such databases is a critical step in the study and improvement of clinical management of specific diseases to reduce their burden and improve human health^7^. The broad need has been widely recognized by regulatory agencies, including the U.S. Food and Drug Administration (FDA) and The European Medicines Agency, by subject-specific societies^6^, and by research consortiums, including the Lifetime Initiative^8^ and The European Institute for Biomedical Imaging Research^9^.

**Fig. 1:**
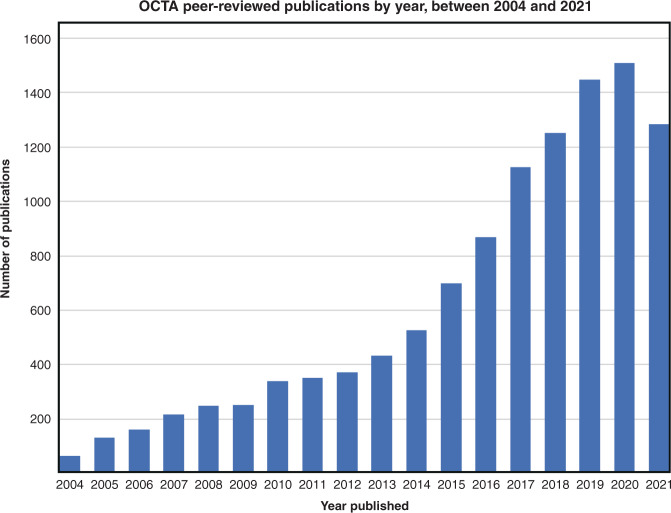
Number of retinal optical coherence tomography angiography peer-reviewed publications by year since 2004. Data sourced: Pubmed, with “optical coherence tomography angiography”, “OCT-angiography” and “retina” as the search key words. Data retrieved on 28 January 2022

In this paper, we set out and make the case for the steps needed to standardize optical coherence tomography angiography (OCTA) imaging of the retinal microvasculature. Through review of imaging protocols, data analysis methods and metrics, reporting of findings, and clinical practice of retinal OCTA, we identify and offer guidance on the standards needed, as well as identify areas requiring further investigation before standards can emerge. The imaging and quantitative analysis of retinal microvasculature are not only important for the study, diagnosis, monitoring, and guidance of treatment of ocular conditions^10^, such as age-related macular degeneration, diabetic retinopathy, vascular occlusion, and glaucoma, but also for systemic conditions, such as cardiometabolic and neurodegenerative diseases^11^. OCTA is an emerging technique attractive to clinicians as it is quickly carried out, non-invasive—not requiring administration of dye—and, distinctively, can provide images of the vascular network from different retinal depths^5^. OCTA derives from optical coherence tomography (OCT), which has proven to be a game changer in ophthalmic care and become the most performed imaging procedure in ophthalmology^12,13^. OCTA has not yet become a standard imaging tool in ophthalmic care, but the field is growing strongly. A continuing increase in the number of publications year on year (Fig. 1) highlights the importance of OCTA to the scientific and clinical research communities and makes it an ideal case study on how to introduce standards in biomedical optics more generally.

The importance of standardization of imaging data in eye healthcare has recently been highlighted by the American Academy of Ophthalmology: “*Standardization would advance the needs and interests of ophthalmologists, their patients, and the quality of clinical care by promoting interoperability, enabling the creation of comprehensive datasets for research and big data analyses, and developing algorithms for machine learning and artificial intelligence”*^14^. As well, standardization has been discussed in the specific context of OCTA^15^ and there have been initial steps taken toward harmonization of the nomenclature and reporting of findings for common retinal diseases^5,16–19^. We build on the evidence presented in these and other previous non-OCTA initiatives^4,5,7,18,20–23^ to advocate clear, rigorous, and easy-to-apply terminology and procedures for physicians, researchers, and OCTA device manufacturers toward the standardization of both technical and clinical aspects of retinal OCTA.

We first set the scene by briefly discussing the retina and its blood supply, the origins and technical background of OCTA, and its application in clinical practice. We then review current imaging protocols, data analysis methods, metrics, and clinical practice. We identify existing discrepancies and inconsistencies and, where possible, propose minimum standards for image acquisition, analysis, and reporting of findings. Where not possible, we identify areas requiring further investigation. We do not review OCTA signal generation and processing, as these topics have been well covered in other reviews^10,24–27^. Due to space limitations and its distinctiveness, we also largely exclude choroidal circulation.

We hope that this paper will contribute to global momentum for the establishment of guidelines for standardized and transparent data collection, analysis, and reporting in OCTA and in biomedical optics more generally. For OCTA, such standards will accelerate scientific discovery and its establishment as a standard of care in clinical medicine.

## The retina, its blood supply, and clinical significance

**Fig. 2:**
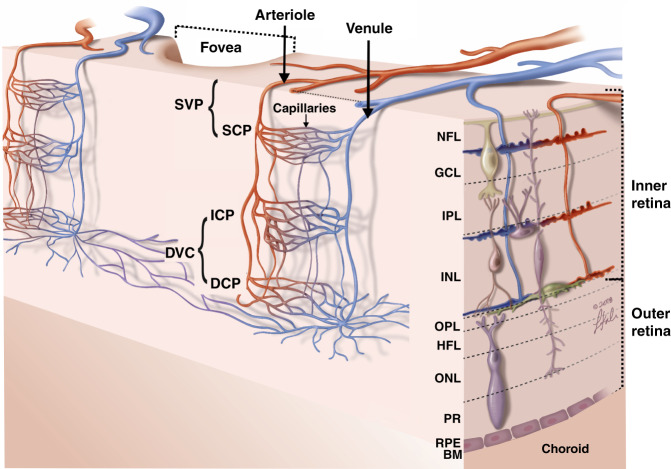
Drawing of retinal circulation. Light impinges on the retina from above. SVP (superficial vascular plexus) comprises superficial arterioles and venules and SCP (superficial capillary plexus); DVC (deep vascular complex) includes ICP (intermediate capillary plexus) and DCP (deep capillary plexus). SCP, ICP, and DCP are thin vascular complexes that are connected to one another and receive the arteriolar supply (red) from SVP arterioles and drain (blue) to SVP venules. NFL, nerve fiber layer; GCL, ganglion cell layer; IPL, inner plexiform layer; INL, inner nuclear layer; OPL, outer plexiform layer; HFL, Henle fiber layer; ONL, outer nuclear layer; PR, photoreceptors; RPE, retinal pigment epithelium. The Bruch’s membrane (BM) is just below the RPE. The inner retina is defined as the NFL to OPL, and the outer retina (which contains no vessels) as the HFL, ONL, PR, and RPE. Not shown: in the peripapillary retina (near the optic disc, not shown here but presented in Fig. 3), an additional vascular plexus is present: RPCP (radial peripapillary capillary plexus). RPCP and SVP together are commonly known as the SVC (superficial vascular complex). Adapted with permission from Nesper and Fawzi^189^

The retina is a semi-transparent neural tissue that lines the internal rear (posterior) surface of the eye. Its function is to generate visual perception by converting the projected image of the world created by the optics of the eye into electrical signals that are transmitted via the optic nerve to the brain. Despite having an average thickness of only 300 µm, the retina has an enormous capacity for parallel processing of complex visual signals, due to the multilayered and pixelated organization of retinal neurons^28^. A schematic of the retinal blood supply and fovea is shown in Fig. 2. The foveola, circa 350 μm in diameter, is the avascular central region of the retina with maximum visual acuity. The high information processing demands of visual perception places a high energy demand on the retina^29,30^ and, therefore, maintenance of adequate oxygen supply and waste exchange within the retina are critical for its function. Oxygen is delivered via a combination of retinal and choroidal microcirculation^29,31^. The inner retinal microcirculation (Fig. 2) is fed from arterioles, flows to capillaries in three layers, and drains through venules; with all vessels having a diameter of 100 µm or less^32^. The microvascular architecture of the inner retina is comparatively sparse transversally and axially because light must pass through vascular regions to the photoreceptors in the outer retina and vessels are highly scattering and disrupt image formation^32^.

The macula, defined as the central retinal region of circa 5.5 mm in diameter, is responsible for high-resolution and color vision, also contains the fovea. Mean capillary flow velocity in the macula is in the range 1.4–3.3 mm/s, which is comparable to that of the brain and higher than in the gut mesentery, for example, for which the mean velocity is 0.8 mm/s^33^. The velocity ranges of blood flow across the whole retina are 1.8–7.2 mm/s in the retinal arterioles and venules and 0.2–3.3 mm/s in the retinal capillaries^34^. By comparison, the ranges for choroidal arterioles and venules and choroidal capillaries (choriocapillaris) are 5.5–12 mm/s and 0.3–3.6 mm/s, respectively.

**Fig. 3:**
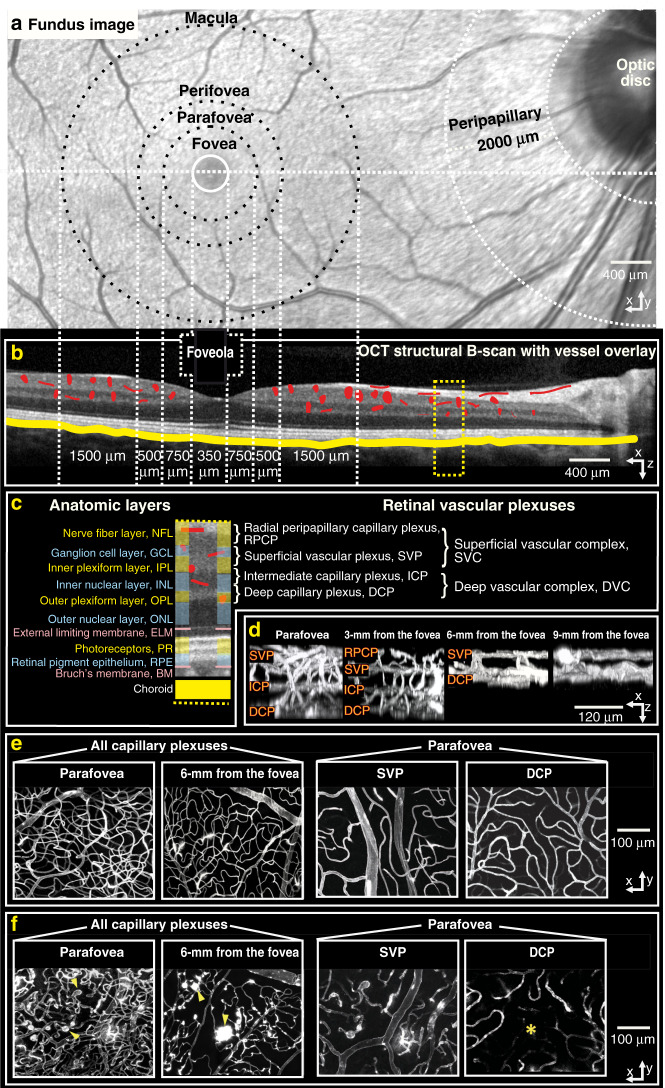
Retinal microvasculature in practice. **a** Fundus camera image from a healthy volunteer with marked retinal sections^190^; **b** Matched OCT structural B-scan with detected vessel overlay; red—retinal and yellow—choroidal vessels; **c** anatomic localization on the OCT image of the retinal vascular plexuses and their names; **d** Confocal microscopy ex vivo showing in-depth organization of retinal vasculature at different transverse distances (eccentricities) from the fovea; **e**, **f** Confocal microscopy fundus view of retinal vasculature at different eccentricities (left) and different plexuses from the parafoveal region (right). Images in **d**, **e** and **f** obtained from deceased human donor eyes perfused with fluorescent contrast agent without retinal disease (**d**, **e**) and with diabetic retinopathy (**f**) Yellow arrows in **f** indicate microaneurysms and the yellow star marks an area of impaired capillary perfusion. Reduced vessel density in the subject with diabetic retinopathy is observed. **d**, **e**, **f** Adapted with permission from An et al.^37^

The outer retinal layers (Fig. 2) are avascular and depend on diffusion from the inner retinal and choroidal microcirculations for metabolic support^32^. The architecture, function, and hemodynamics of the microcirculation in the inner retinal layers vary between layers and across retinal locations to meet the distinct and varied metabolic demands of the local cellular environment (Fig. 3)^30,32,35^. The high overall oxygen demands of the retina and relatively sparse nature of its microvasculature require dynamic, well-regulated blood flow. In contrast, the choroidal vasculature is denser and lacks this regulatory ability^30^. Figure 3 further illustrates the retinal microvascular network. Human volunteer fundus photography is correlated with OCT cross-sectional imaging. As well, confocal microscopy shows the retinal microvascular network at different depths and eccentricities of perfused donor retinas (ex vivo).

The visualization and characterization of microvascular networks in different spatial domains of the retina is vitally important for improving the understanding, classification, and staging of a wide range of common diseases with a vascular component (either as cause or effect)^36^, including age-related macular degeneration (AMD), diabetic retinopathy, vascular occlusion, coronary heart disease, and Alzheimer’s disease. Even small reductions in blood flow have been shown to have deleterious effects on retinal function^30^. Vessel density reduction in diabetic retinopathy occurs in multiple retinal locations but most commonly in the deep capillary plexus^37^. An abnormal superficial vascular complex in the optic nerve can be present in glaucoma, whereas, an abnormal macular deep capillary plexus can be present in retinitis pigmentosa^38^. The peripapillary circulation region, located at the margin of the optic nerve head, is a critical site in the pathogenesis of retinal vein occlusion, optic disc rim (i.e., Drance) hemorrhage, and glaucoma^39,40^. Vessel density reduction may be present in all retinal and choroidal layers in coronary heart disease^41^ and present in the superficial vascular complex in Alzheimer’disease^42^.

## Overview of optical coherence tomography angiography and retinal applications

We have identified over 300 clinical trials registered at www.clinicaltrials.gov evaluating OCTA as a diagnostic tool for screening of retinal and choroidal dysfunction in ocular and systemic diseases. In its most common configuration, OCT captures, without contrast agent, a volumetric image dependent on the scattering properties of the tissue by transversally scanning a focused beam and collecting the depth-encoded scattering profile (termed A-scan) through (spectral-domain) low-coherence interferometry. Transverse scanning captures a line of A-scans termed a B-scan, and defines the fast (*x*) axis. Slower scanning in the orthogonal transverse direction (slow *y* axis) then completes a volume scan. OCTA enables volumetric visualization of retinal vasculature from the motion contrast generated mainly by scattering from erythrocytes (red blood cells) in flowing blood causing the speckled OCT signal to fluctuate. OCTA images are extracted by analysis of the time-varying OCT signal (intensity and/or phase) between acquisitions, most commonly cross-sectional scans (B-scans) at the same or closely adjacent locations^38^. The processed volume created by the stack of OCTA B-scans is usually presented as a maximum intensity projection image in the transverse plane over a specified depth range.

The use of fluctuating optical speckle to characterize tissue substantially predates OCTA; even its use in OCT dates to the 1990s. Its use in characterizing flow dates back to at least Barton and Stromski in 2005^43^ followed by the seminal work of Makita et al.^44^, Wang et al.^45^, and Fingler et al.^46,47^, in developing and applying OCTA for retinal imaging. Although, Makita et al. introduced the term “optical coherence angiography” and Wang et al. “optical angiography”, the acronym OCTA has become widely adopted^48^. As yet, OCTA methods do not allow accurate blood flow quantification^49^, but there are ongoing attempts to solve this difficult problem^50^. The first commercial OCTA instrument, the spectral-domain (SD) OCTA from Optovue, was approved by the FDA in 2015^51^. In the same year, the first commercial swept-source (SS) OCTA system was introduced by Topcon^10^. Currently established commercial OCTA device suppliers include Optovue, Topcon, Heidelberg Engineering, Carl Zeiss Meditech, Canon, Optopol, and Nidek^25^.

Commercial instruments usually acquire 70,000–100,000 A-scans per second using a light source centered at a wavelength of either 840 nm or 1050 nm, for SD-OCTA or SS-OCTA, respectively, with an interscan time (Δ*T*, the interval between B-scans) of 4–5 ms, and achieving optical axial resolution (product of physical resolution and group refractive index of the medium) of 5–10 µm and transverse resolution of circa 20 µm; these resolutions are the full-width at half-maximum in tissue^10^. Research instruments can operate at substantially higher acquisition speeds, up to megahertz A-scan rates^52^, Δ*T* of <1.5 ms^10^, and resolutions as high as circa 3 µm^53^.

In principle, more than two B-scans can be recorded at a single location and used to generate multiple interscan times, with associated data storage and processing overhead^54^. Commercial instruments usually collect between two and four B-scans at the same location^10^. The value of the interscan time determines the maximum flow velocity capable of generating speckle contrast and, therefore, which vessels are visible in the OCTA image^10,34^. As discussed, blood flow velocity in the retinal circulation can be anywhere in the range 0.2–7 mm/s. For retinal vessels oriented perpendicular to the imaging beam, the maximum distinguishable velocity can be found by dividing the transverse resolution by Δ*T*; therefore, for Δ*T* = 1.5 ms and Δ*T* = 5 ms and transverse resolution of 20 µm, maximum speeds are 13 mm/s and 4 mm/s, respectively. These values are reduced for non-perpendicular vessels. On the other hand, the longer the interscan time, the better the sensitivity to low flow velocities, as more speckle decorrelation occurs between scans; thus, there is a trade-off to be struck. There remains strong continuing interest in quantifying blood flow with OCTA. Low decorrelation values are proportional to flow velocity but full decorrelation leads to a saturation velocity value. Thus, there is a further trade-off in selection of interscan times that is outside of the current scope of our discussion^10^.

The resolution of the OCTA images—in all three spatial dimensions—represents a trade-off between visibility of vessels, their accurate representation, and sensitivity to motion artifact. The standard transverse resolution of ∼20 µm of commercial systems enables imaging over several hundred micrometers in axial depth, typically covering an angular field of view (FOV) of between 10 × 10 and 30 × 30 degrees that corresponds to a linear FOV of between 3 × 3 mm and 9 × 9 mm on the retinal surface of a typical eye. (The exact correspondence depends on the subject: correction of transverse image magnification error is further discussed below.) At the standard transverse resolution, the caliber of some capillaries appears wider than their true width^38^. High transverse resolution (below 10 µm, only available from research systems) helps to resolve small features of the microvascular network^53^, which is especially important for visualizing the densely packed choriocapillaris. However, increased resolution is usually obtained over a reduced imaging depth and FOV and makes the measurement more sensitive to motion artifacts that degrade the quality of OCTA images. Any eye movement sufficient to cause OCT speckle to decorrelate between acquisitions (as a rule of thumb, requiring motion with magnitude on the order of the resolution) will generate OCTA contrast. Bulk motion artifact correction allows efficient removal of axial eye motion artifact; transverse eye motion correction is not so easily achievable.

There have been many algorithms introduced to generate OCTA maps and these can be divided into phase-, amplitude- and complex-signal methods^24,38,49,55^. Phase-based methods, although capable of generating excellent quality OCTA images^44,46,47,56^, require precise removal of background phase noise caused by eye bulk motion or from system instabilities, which is challenging^57,58^. Therefore, such methods have not yet been applied in commercial instruments. Amplitude-based methods^43,59^ are applied in most commercial instruments (e.g., Optovue, Topcon, and Heidelberg)^38^. Complex-signal-based methods demonstrate high sensitivity to low-velocity flow; however, they are sensitive to eye movements too. Optical microangiography (OMAG) is one such prominent research example^45^ also used commercially by Zeiss^38^.

Additional post-processing can be applied to OCTA datasets to enhance the quality of the final angiogram. The split-spectrum amplitude-decorrelation angiography (SSADA) algorithm was the first to be used in a commercial instrument (Optovue)^10^. SSADA splits the OCT optical frequency spectrum into bins to reconstruct multiple lower axial resolution images to reduce the effects of axial eye motion, before recombining them to regain the signal-to-noise ratio^60^. As well, averaging of multiple OCTA datasets^61^ produces valuable improvements in OCTA image quality, as we discuss below.

**Fig. 4:**
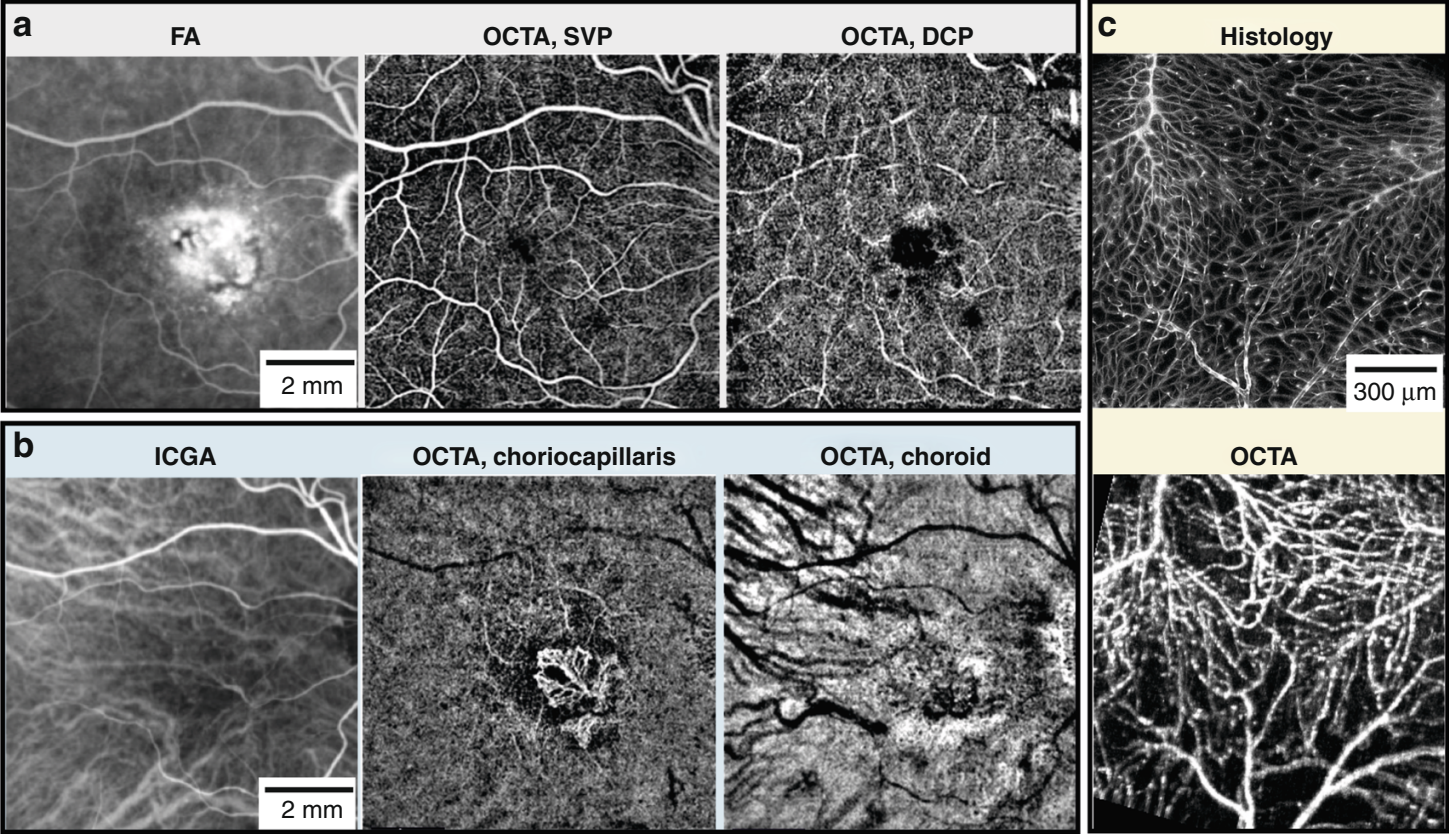
Comparison of OCTA, fluorescein angiography (FA), indocyanine green angiography (ICGA), and histology. **a**, **b** FA, ICGA, and OCTA segmented to superficial vascular plexus (SVP), deep capillary plexus (DCP), choriocapillaris, and choroid, from a patient with choroidal neovascularization secondary to age-related macular degeneration. **a** FA shows leakage of dye obscuring details of the fine blood vessels within the choroidal neovascularization that can be more clearly seen in OCTA at the level of choriocapillaris than ICGA in (**b**). **c** Confocal microscopy and OCTA from an isolated perfused porcine eye ex vivo. For the OCTA image acquisition, the whole porcine eyeball was used, and the retinal vasculature was perfused using red blood cells. After the OCTA experiment, retinal vasculature histology was performed. The retina was perfused with fluorescein as a contrast agent, and the eyeball was cut open, flat-mounted and imaged using confocal scanning laser microscopy. Both images are maximum intensity projections from the full retinal thickness. **c** Adapted with permission from Yu et al.^68^

The current commonly used clinical angiography techniques are fluorescein angiography (FA) for the retina and indocyanine green angiography (ICGA) for the choroid. OCTA enjoys the advantage over these that it does not require a contrast agent; thereby, avoiding the rare adverse patient reactions to the dye^62^. As well, OCTA acquisition times can be as short as a few seconds compared to the 5–10 min required (after dye administration) to capture the time-lapse sequences required in FA/ICGA^58^. Perhaps the greatest advantage of OCTA is the resolution in depth from the volumetric images not available from the two-dimensional FA/ICGA images. In particular, OCTA provides better visualization of the deep capillary plexus and choroidal vasculature compared with FA and ICGA^63,64^ (Fig. 4a, b). Conventional FA/ICGA images 30–50 degrees of the retina, however, ultra-wide-field FA/ICGA imaging systems have been developed to image the retina spanning up to 200 degrees^65^. As mentioned, typical clinical OCTA angular FOVs cover 10–30 degrees. Lab-based OCTA instruments have achieved up to 70 degrees in a single measurement^66^ and 100-degrees by applying a montage scanning protocol^67^. Whilst FOVs for FA/ICGA and OCTA have become very comparable, OCTA cannot visualize leakage or vascular permeability important for clinical diagnosis and management^10^.

OCTA demonstrates good correlation with histology (Fig. 4c)^49^ but tends to yield larger vessel area densities and larger capillary diameters^68^, which has been confirmed by retinal vessel diameter measurements comparing SS-OCT to adaptive-optics ophthalmoscopy^69^.

OCTA has already enabled novel discoveries in ophthalmology^55,58,70,71^. For example, OCTA has provided insight into the relative importance of the superficial versus deep capillary plexuses in macular telangiectasia^72^ and diabetic retinopathy^73^. Promising early-stage lab-based research includes the study of retinal vascular function, including monitoring of pulse wave velocity^74^, blood flow heterogeneity^32^, and response to external stimulation^75^. Vascular pulsatility is a field of the future for OCTA, with early work in the retina based on Doppler OCT, and early work using OCTA directed at skin imaging^76,77^. Looking ahead, OCTA may also be beneficial for neurological research^51,78^ to advance understanding of pathophysiology of multiple sclerosis^79^, Alzheimer’s disease^42,80–84^, various optic neuropathies^51^, and diagnosis of cerebral small vessel disease^85^.

## Review and standardization of OCTA imaging protocols

In this section, we discuss key OCTA scanning protocols and imaging parameters to support standardizing image collection procedures and their reporting. We include the important emerging area of widefield OCTA and pay particular attention to the harmonization of terminology.

### Scanning field of view and sampling density

We first consider the effect of the FOV on raster scanning, which is commonly used in both commercial and non-commercial instruments^86^.

**Fig. 5:**
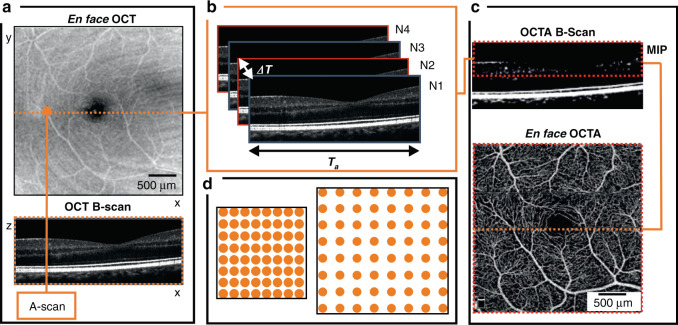
Simplified schematic of OCTA scanning protocol. **a** A raster scanning protocol is applied to visualize blood vessels; A-scans per B-scan sets *x*-axis sampling density; B scans per volume sets *y*-axis sampling density. **b** Four repeated B-scans at one *y*-location are used to create an OCTA B-scan; the procedure is repeated for successive positions along the *y*-axis; the final number of OCTA B-scans impacts on sampling density along *y*-axis; Δ*T*—interscan time; *T*_*a*_—acquisition time. **c** Maximum intensity projection (MIP) is applied to OCTA B-scan over depth range of interest (where vessels are located) to generate one line of the en face OCTA image. **d** Illustration of how the same number of sampling points is distributed for smaller and larger imaging areas

The OCTA image construction process is illustrated in Fig. 5. The linear scan length defines the FOV and the combination of scan length, number of A-scans per B-scan, and number of OCTA B-scans enables calculation of the transverse scanning density (usually termed sampling density) in the *x* and *y* directions. Sampling density is calculated as: *ρ*_*x,y*_ = Δ*x*/s*can separation*_*x,y*_, where Δ*x* is the OCT transverse resolution, *scan separation*_*x*,*y*_ is the scan length divided by the number of samples (either number of A-scans along the *x*-axis or OCTA B-scans along the *y*-axis). A sampling density of 2 represents the Nyquist limit. A value of 2.5 or higher would ideally be used to provide a margin to accommodate non-ideal factors but, in practice, commercial systems routinely use sub-Nyquist sampling.

In practice, the choice of FOV and sampling density requires compromise. A larger FOV is often associated with lower sampling density to maintain the same OCTA volume acquisition time. The lower sampling density results in a lower image resolution and detection of fewer smaller vessels. Analysis of images produced at lower sampling density leads to lower image-grading agreement and reduced vessel density measures^55,87–89^. Reduced sampling density may additionally affect the accuracy of vessel diameter assessment, already biased by the system’s transverse resolution, at all FOVs^69^.

3 × 3-mm FOV is often chosen as a good compromise to maintain adequate sampling density and image acquisition time. For example, for OCTA parameters of 4 B-scans per *y* location, 100,000 A-lines per second and 300 × 300 samples per FOV, total acquisition time is 4 s. For lower numbers of B-scans at each *y* location, this time can be further reduced, but at the cost of OCTA image quality.

**Fig. 6:**
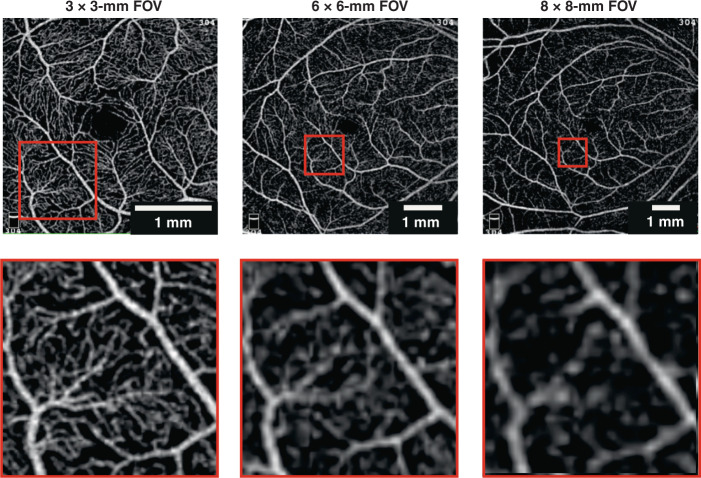
Comparison of OCTA images versus FOV and sampling density. All images acquired with AngioVue. First row shows original images. Second row shows zoomed images of the same 1 × 1-mm region of interest with transverse scanning density (left to right) of 2, 1, and 0.85, respectively, strongly suggesting only the images in the first column are of sufficient quality for quantification purposes

Not all commercial platforms keep sampling density constant as FOV changes. For instance, RTVue XR Avanti (Optovue, Inc., Fremont, CA, USA) offers retinal scanning protocols: either 3 × 3-mm or 6 × 6-mm FOV with fixed 304 × 304 A-scan sampling, sampling densities of 2 and 1 (sub-Nyquist), respectively, for a transverse resolution of circa 20 µm. Plex Elite 9000 (Carl Zeiss Meditec, Dublin, CA, USA) provides a 3 × 3-mm FOV with 300 × 300 A-scans and a 6 × 6-mm FOV with 500 × 500 A-scans, partially compensating sampling density as the FOV increases, at 2 and then 1.7, also for circa 20 µm transverse resolution. Due to the limitation on acquisition speed of current OCTA systems, sampling densities of 1.7 and above represent a reasonable compromise for gross OCTA image quantification. The importance of adequate sampling density is highlighted in Fig. 6.

We recommend reporting the sampling density (currently rarely reported) in addition to the transverse resolution and image size, to enable interpretation of all analyses, especially quantitative ones.

### Widefield OCTA

Widefield OCTA imaging extends the raster scanning protocol to achieve much larger FOVs. Images are captured as single shot, as demonstrated, e.g., by Polans^66^ and Wei^90^, or by mosaicking/montaging of scans, as shown by Hendargo^91^ and Zhang^67^. A wide variety of image sizes and montages have been reported, from a single scan of 12 × 12 mm to montage scans comprising: two 15 × 9-mm scans; five scans of 12 × 12-mm each; and five scans of 6 × 10-mm each^5^. The term “ultra-widefield” OCTA has been introduced to describe 100-degree angular FOV OCTA imaging comprising a 4 × 4 grid of 6 × 6-mm scans^67^. Recently, three slightly different definitions have been proposed for widefield and ultra-wide-wield OCTA by Munk et al.^5^, Choudhry et al.^92^, and Pichi et al.^18^. Based on their investigations and discussions, we recommend defining widefield and ultra-widefield OCTA, independent of the use of montaging, as producing an image in the angular FOV range 70–110 degrees and above 110 degrees, respectively; this is aligned with definitions commonly applied in retinal fundus photography^93^.

## Review and standardization of OCTA data analysis procedures and terminology

In this section, we discuss a wide range of OCTA data analysis procedures and terminology applied to collected OCTA datasets to make them ready for clinical evaluation. We include OCTA image processing, retinal layer segmentation and definition, image artifacts, image averaging, image quality indicators and classification, and correction of transverse image magnification error.

### En face OCTA image processing

En face OCTA maps (also termed C-scans) can be created from the volumetric OCTA dataset from any retinal layer of interest. These maps are usually calculated by mean projection (the average value of voxels at a given *x*, *y* position over a given depth range) or maximum intensity projection (MIP, the maximum value voxel in depth). The MIP, in effect, ignores the overlap of multiple vessels or projection (shadow) artifacts (described below), thereby, providing a cleaner angiogram^38,94^.

Several processing steps are commonly applied to en face OCTA images before quantitative analysis is undertaken^95^, including image filtering (e.g., using a Frangi “vesselness” filter to improve vessel visualization)^96^, binarization (e.g., using Otsu, global, fuzzy, or other thresholding methods)^97–99^, and image skeletonization. As well, the correction of vessel discontinuity in the OCTA binary image, by filling the gaps between vessel segments, has been reported^23^. Because of the fine line between appropriate interpolation and inappropriate image manipulation, we recommend not to apply this procedure to avoid ambiguous manipulation of images but, if applied, a rigorous description of steps taken should be given. The recent review papers by Tan et al.^95^ and Meilburger et al.^100^ summarize the most common segmentation methods applied to OCTA images.

Hong et al. have demonstrated the effect of vessel enhancement filters on the repeatability of vessel area density^101^. Rabiolo et al. and Mehta et al. have independently demonstrated that differences in binarization thresholding methodologies influence the quantification of OCTA metrics^97,99^. Linderman et al. have shown that the choice of segmentation method affects repeatability and accuracy of foveal avascular zone (FAZ) assessment^102^. Corvi et al. reported that the measured values of OCTA metrics significantly differ among OCTA instruments^103^. These studies highlight the need for consensus within the community regarding the optimum pipeline for OCTA image processing^95^. More comparative studies, such as those of Gorczynska et al.^104^, Giarratano et al.^105^, and Dadkhah et al.^106^, are needed to benchmark the available algorithms and select the best candidates for image processing and segmentation. Better open access analysis code and better datasets of the same individual imaged on several platforms, to use as a baseline or benchmark, would be very helpful in realizing this objective. Well-defined, optimized pipelines for OCTA en face image processing and analysis will be critical precursors for the successful conduct of large-scale multicenter studies to pave the way for the development and validation of OCTA-based biomarkers^21^.

### Retinal layer segmentation and definition

Several algorithms have been introduced enabling automated segmentation of retinal layers based on computer vision^91,107–109^ and deep learning^110,111^. Commercial instruments usually have pre-set layers of interest segmented through an automated process; however, expert manual correction is often required, especially in diseased retinas^58^. More work is still required to find better, more universal methods for automated segmentation of OCTA images that do not require manual intervention^49,112,113^. The pre-set layers and even the definition of layers vary slightly between commercial instruments, which also holds true for non-commercial instruments and studies, and will likely cause bias in quantifying OCTA metrics^17^.

Campbell et al. have introduced terminology for the retinal circulation^114^ aligned to current knowledge of retinal anatomy gained from histological studies^28,115,116^. We note that this terminology has been recently further updated by Hormel et al., who have proposed replacing the term radial peripapillary capillary plexus with nerve fiber layer plexus (NFLP) and the term superficial vascular plexus with ganglion cell layer plexus (GCLP)^38^. Since Campbell et al.’s terminology has already been widely applied, we recommend adopting their layer names and boundary definitions (Fig. 3c)^114^, as described below, and reserving consideration of further updates. Such consistency will enable generation of a critical mass of studies of the same microvascular networks for which comparison between studies will be meaningful.

We summarize Campbell et al.’s definitions below (see Figs. 2 and 3c).

*Superficial vascular plexus (SVP)*—defined as the inner 80% of the ganglion cell complex (GCC) that includes the nerve fiber layer, ganglion cell layer and inner plexiform layer^114^. Characterized by a dense irregular lattice work of vessels composed of larger arteries, arterioles, capillaries, venules, and veins^28,117^.

*Superficial capillary plexus (SCP)*—that part of the SVP comprising only capillaries; capillaries in this network demonstrate looping.

*Intermediate capillary plexus (ICP)*—comprises capillaries composed of vertical and oblique segments and located^28^ within the outer 20% of the GCC and the inner 50% of the inner nuclear layer (INL).

*Deep capillary plexus (DCP)*—comprises capillaries arranged in a laminar plane^28^ and located within the outer 50% of the inner nuclear layer (INL) and the outer plexiform layer (OPL) (includes Henle’s fiber layer; not shown in Fig. 3 but visualized in Fig. 2).

*Deep vascular complex (DVC)*—is composed of the intermediate capillary plexus (ICP) and the deep capillary plexus (DCP).

*Radial peripapillary capillary plexus (RPCP)*—is present in the peripapillary retina and characterized by long capillary segments predominantly oriented parallel to the nerve fiber layer axons. RPCP plays an important role in supplying the densely packed nerve fiber layer^28,117^.

*Superficial vascular complex (SVC)*—groups the RPCP and SVP.

### Image artifacts

OCTA suffers from various image artifacts that impact image quality and interpretation and are important to avoid or recognize and mitigate. Artifacts can be caused by the nature of OCTA data collection and processing, eye movement, and properties of the eye and light-matter interaction. There are many reports seeking to introduce standardized terms and definitions of artifacts^86^. Most often, artifacts have been defined and described as:

- *segmentation artifacts* caused by improper segmentation of retinal layers by automated software^118^;
- *motion artifacts* caused by relative eye-instrument movement during image acquisition^118,119^;
- *blink artifacts* caused by the patient blinking during imaging;
- *shadow artifacts* caused by OCT signal attenuation during imaging and appearing as areas of decreased flow information in the image and due to, e.g., large vessels, cataracts, vitreous floaters, and pathology within the retina such as drusen^120^;
- field-dependent *ocular aberrations* that can introduce variable image quality in wide-field OCTA images and artifacts, such as double rendering of vasculature^66^; and
- *projection artifacts*, also known as decorrelation tails, which are particularly challenging and universal to all OCTA devices, as they unavoidably result from the light-matter interaction with vessels^38,86,121^. They cause the projection of the more superficial vascular network into the deeper networks. Without the removal of projection artifacts, it is difficult to obtain accurate metrics describing the microvascular network below the SVP.

There have been various algorithms introduced for removing projection artifacts in research, such as slab subtraction (operating on en face images only)^122,123^ and projection-resolved OCTA algorithms (operating on en face and cross-sectional images)^38,124,125^. Commercial instruments now also include proprietary projection artifact-removal algorithms. Projection-resolved OCTA algorithms generally produce better results than slab subtraction methods, however, can also produce images containing residual artifacts^121^. It is important to report in publications which algorithm has been used to mitigate projection artifacts, whether residual artifacts remain and, if so, how they impact on the assessment of the results. We advocate the use of the artifact terminology described here and strongly encourage the incorporation of projection artifact mitigation into both research and clinical OCTA imaging products. Further work is required to compare and select the best of the available algorithms.

### En face image averaging

Averaging of multiple en face OCTA images (projections) improves image quality^126–128^ by reducing noise that could otherwise be misinterpreted as flow, by improving the connectivity of vessels^98,129,130^, and by making the vessels sharper and thinner^127^. Averaging modifies quantitative automated measurements; for example, leading to decreased vessel density^98,127,129,130^. It may also improve microaneurysm detection^131^ and diagnostic accuracy of metrics^127^. Overall, averaging enhances the visualization of vessels which makes qualitative and quantitative assessment of OCTA images more reliable^132^.

Uji et al. reported that an average of a minimum of six en face images was needed for ideal binarization of macular OCTA images from the deep capillary plexus and two images were needed from the superficial vessel plexus when using the Zeiss Cirrus 5000 (Carl Zeiss Meditec, Dublin, CA, USA)^98^. Nelson reported that averaging three en face images significantly improved vessel visualization of the radial peripapillary capillary layer when using the Zeiss Cirrus 5000^127^. Schmidt et al. reported the need for averaging five OCTA en face images with the Optovue instrument to improve vessel segmentation, visibility and continuity, and final OCTA metrics^133^. Mo et al. have shown that for the Optovue instrument averaging 5–9 en face OCTA images significantly improves SNR and averaging 4–7 OCTA frames improves skeletonization metrics^128^. Furthermore, Nelson et al. has demonstrated that OCTA averaging has improved diagnostic accuracy of vessel area density and significantly improved vessel skeleton density assessment of glaucoma; indicating that the averaging procedure may help better identify clinically valuable microvascular network biomarkers^127^.

Averaging typically requires multiple volumetric scans to be collected which may lengthen total patient time in the clinic, which is an imposition on individuals undergoing OCTA examinations and a clinical overhead. More systematic studies on averaging are required before recommendations on standards can be made. Until then, we recommend not comparing metrics obtained on single images with those from averaged images as it has been demonstrated that they are not equivalent^98,133^.

### OCTA image quality indicators and classification

Yu et al. have demonstrated that vessel density measurements correlate with OCTA image quality^134^. It is clear that standards for image quality are needed. Automated image assessment procedures are under development for OCTA image interpretation, seeking to distinguish both good and poor image quality, and to identify the type and severity of disease. Automated OCTA image quality indicators have been introduced by some OCTA manufacturers, e.g., Optovue’s scan quality index (SQI). However, consensus on the choice of threshold values for acceptable image quality has not been reached. For example, Optovue has recommended that an OCTA image with an SQI below 6 should be excluded from analysis. Ali et al. have shown that an SQI of 7 or above should be considered for the Optovue OCTA and is insufficient on its own—visual inspection or image-grading should still be applied^135^. Wang et al.^85^ and Mirshahi et al.^136^ accept image quality based on SQI above 5 for the Optovue OCTA. Lim et al. have recommended only using OCTA images with “Zeiss signal strength” of at least 9 for the Zeiss Cirrus 5000 with an AngioPlex OCTA (Carl Zeiss Meditec, Dublin, CA, USA) to obtain reliable microvascular metrics, whereas, the manufacturer recommends a threshold of 6^137^.

Lauermann et al. reported a deep learning algorithm to classify images as sufficient or insufficient^138^. Automated methods have also been investigated to identify the type and severity of diseases, including diabetic retinopathy^139,140^, AMD^141^, and glaucoma^142^. Overall, much more work is required to demonstrate how algorithms perform with images from different retinal depths and obtained with different OCTA instruments. Alternatively to image quality assessment, methods that are tolerant to a wide range of OCTA image quality could be developed, such as convolutional neural network-based deep learning models^143,144^. There is, as yet, no consensus on the best methods to either assess OCTA image quality or undertake reliable analysis that is “image-quality independent” and, thus, more work on these topics is needed^111^.

### Correction of transverse image magnification error

**Fig. 7:**
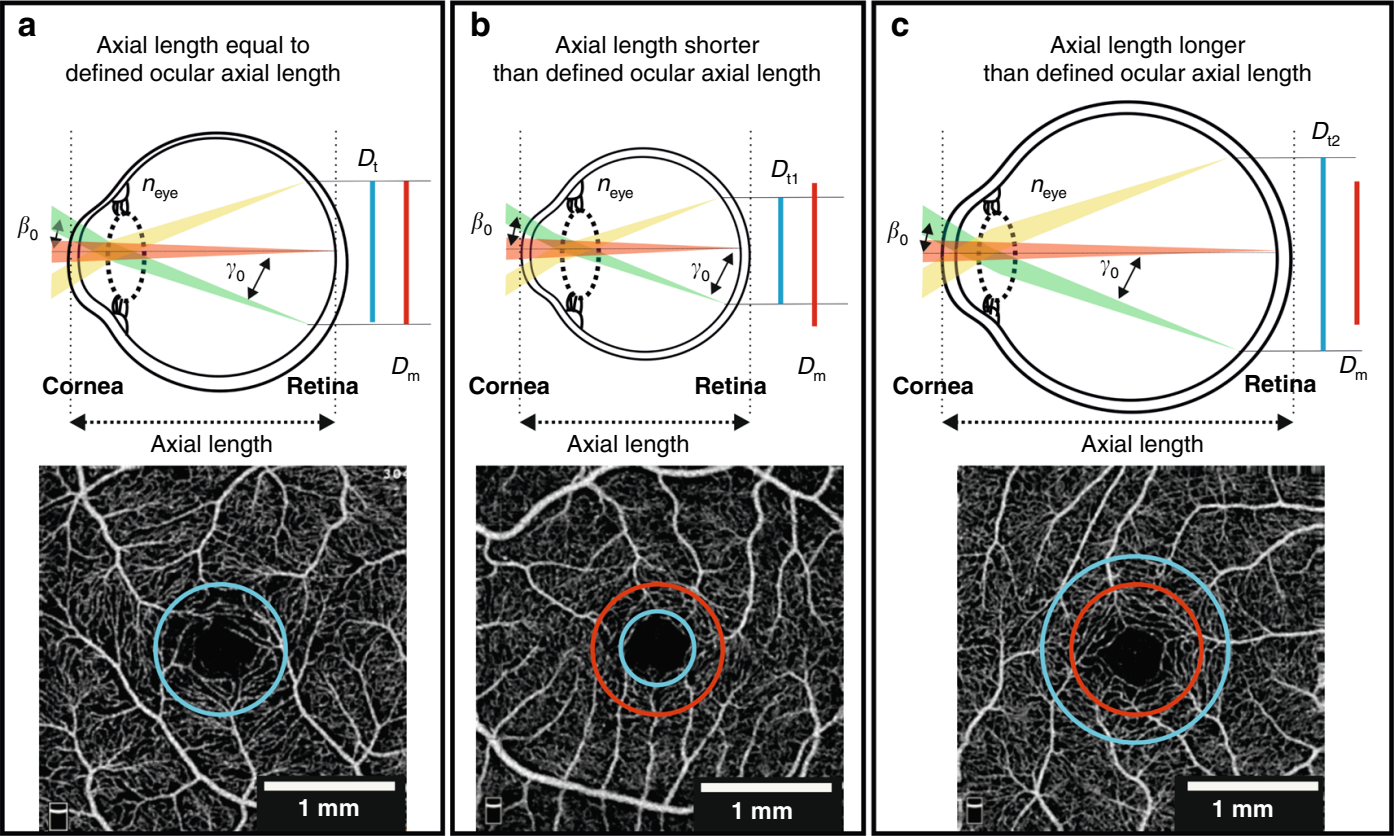
Effect of varying axial length on OCTA scan dimensions. The incident beam is shown for three scan paths (green, red, and yellow; *β*_0_ is the incident angle at the cornea and *γ*_0_ is the angle subtended on the retina); green and yellow paths represent the maximum deviations, and the red path is on axis. Refraction from the scan lens (not shown), cornea, and lens focuses each light path on the retina. **a** Axial length equal to the defined ocular axial length of OCTA instrument so the linear size of the measured retinal diameter (*D*_*m*_) corresponds to the true retinal diameter (*D*_*t*_). **b** Axial length shorter than defined ocular axial length results in *D*_*t*1_ < *D*_*m*_. **c** Axial length longer than defined ocular axial length results in *D*_*t2*_ > *D*_*m*_. Below each schematic diagram, a visual representation demonstrates how the OCTA instrument would measure a ring of 1 mm diameter (without correction for axial length variation, red circle) in each case versus the true 1 mm diameter (blue ring). Adapted with permission from Sampson et al.^147^

Nominally, the linear OCTA scanning area (expressed in mm^2^) is derived from the calculation of the linear distance on the surface of the retina subtended by a fixed angular FOV using a defined ocular axial length (e.g., for the Optovue RTVue XR Avanti system, this length is fixed at 23.95 mm). Thus, a fixed angular FOV of 10 × 10 degrees will correspond to a 3 × 3-mm FOV only for individuals with an axial length 23.95 mm (Fig. 7). To convert retinal images from angular units, an FOV correction for the variation in real axial length from the reference setting is required^145^. The most common method to correct transverse image magnification error is the Littmann-Bennett method that expresses^146^ the relationship between the measured image diameter *D*_*m*_ and the true diameter *D*_*t*_ as *D*_*t*_ = *p·q·D*_*m*_, where *p* is the magnification factor of the imaging system and *q* that of the eye. The factor *q* can be found from *q* = 0.01306·(axial length−1.82). The factor *p* can be calculated from the Bennett formula setting the axial length so that *D*_*t*_ = *D*_*m*_ thus *p* = 1/*q*. For example, for the RTVue XR Avanti, *p* = 3.46 since the axial length is 23.95 mm.

It would represent a significant advance if more OCTA instrument manufacturers were to make available the axial length for which their system’s scanning protocols have been scaled. Sampson et al. have demonstrated that not compensating for axial length variation in OCTA can result in a relative difference before and after correction of up to 51% in foveal avascular zone (FAZ) area^147^. A similar observation has been made by Linderman et al.^102^. Thus, the impact on vascular parameters of not correcting for axial length is likely to be substantial. However, in a systematic review, Llanas et al. found that only 8% of 509 studies corrected OCTA images for magnification before quantitative analysis^148^.

One factor impeding researchers and clinicians from correcting magnification errors is access to the comparatively expensive biometry device required to measure axial length^149^. Tan et al. have proposed alternatively to correct the FOV by registering the images according to the measured distance between centers of the optic nerve head and fovea, assuming a small individual variation of the distance^150^. However, a study by Rohrschneider has demonstrated considerable variability in the distance between the optic nerve head and the fovea, suggesting this method may not be satisfactory^151^. Recently, Morgan et al. have explored the estimation of axial length from measured refractive error and corneal curvature to support cost-effective myopia management^149^ and have shown these factors offer a good estimate of actual axial length. Therefore, the estimated axial length could potentially be used with the Littmann-Bennett formula to correct image magnification error. However, this potential approach to correction has not yet been investigated. Lal et al. recently empirically demonstrated the need to include both axial length and refractive error when correcting for transverse image magnification error^152^. However, such still more demanding requirements, in measuring more parameters related to eye biometry, run counter to what is required: a simple and low-cost method of correction that avoids measuring axial length. Thus, more research is needed to establish a simple, robust, standard method for image size correction because of its large impact on vessel parameters.

## Review and standardization of metrics for quantitative analysis of the retinal microvascular network architecture and foveal avascular zone

**Fig. 8:**
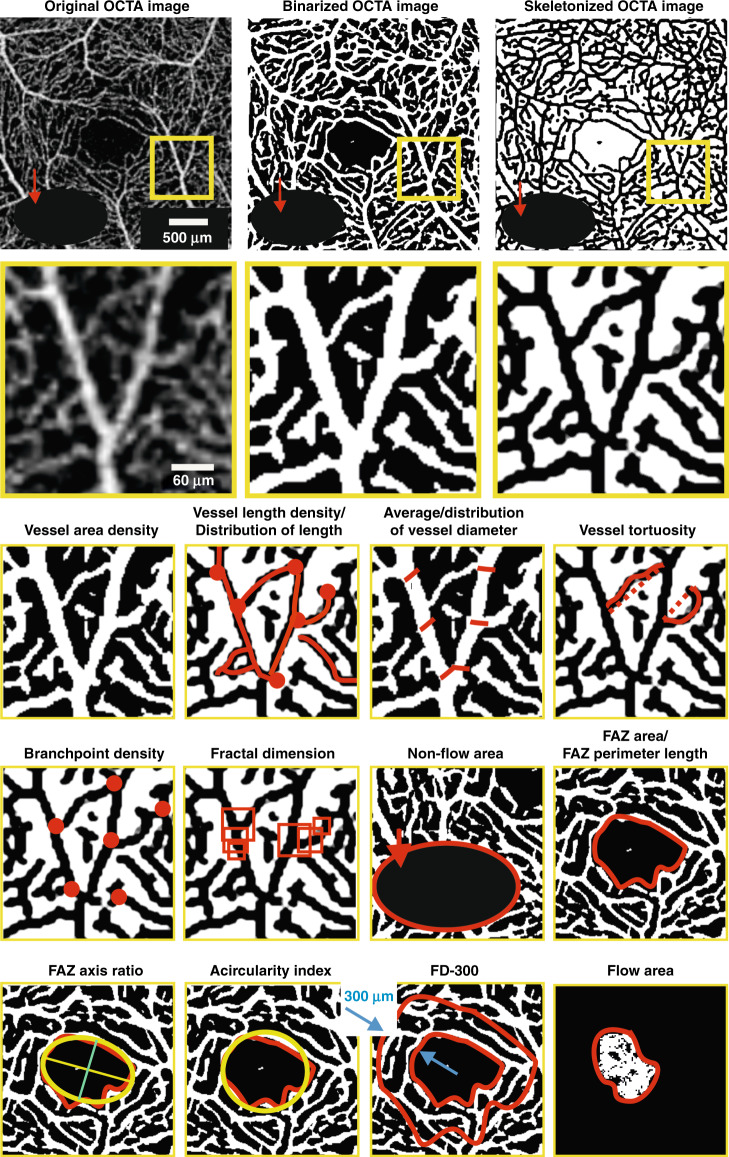
OCTA microvascular metrics. Visual representation of recommended OCTA-based retinal microvascular metrics as defined in Table 1

Microvascular structural and functional biomarkers in the retina can be mapped and quantified using specialized metrics (Table 1, Fig. 8)^33,153^. Since many diseases affect multiple markers, it is important in building a complete picture of a condition to characterize retinal vascular networks using multiple metrics^154^, even if ultimately a minimum set of biomarkers is desirable.

**Table 1:** Recommended metrics for characterization of the retinal microvascular network architecture and characterization of foveal avascular zone based on en face OCTA images

Metric (abbreviation)	Unit	Type of analyzed image	Definition and importance
Vessel area density (VAD)	%	Binarized	Ratio of the area occupied by vessels (white pixels) divided by the total area converted to a percentage. The change in VAD is coupled with changes in vessel length and vessel size. Since the decrease of microvascular perfusion is sometimes accompanied by vessel dilation, VAD alone cannot provide a complete picture of changes in vessel function. Nevertheless, VAD provides the best estimate of real vessel density^154^ and can be used to demonstrate ischemia^167^. *It is recommended that VAD replaces related terms including vessel density and perfusion density*.
Vessel length density (VLD)	%	Skeletonized	Ratio of the total length of the blood vessels to the total area. Each vessel is identified as a single pixel-width line along its centerline. VLD does not consider vessel size and compared to VAD is more sensitive to perfusion changes at the capillary level^154^.
Average, median, and distribution of vessel length	mm	Skeletonized	Lengths of all identified vessel segments along the centerline of the vessel. Network interconnectivity and branching patterns can indicate oxygenation/nutrient delivery dysfunction.
Average, median, and distribution of vessel diameter	µm	Binarized	Diameters of all identified vessel segments. Each diameter found as the distance between edges orthogonal to centerline of blood vessels in the binarized image; each segment should be sampled at least three times. Can inform on dilation, sprouting or vessel regression^153^.
Vessel diameter index (VDI)	pixels	Binarized and skeletonized	Average vessel diameter calculated by dividing VAD by VLD; the unit is pixels and can be converted to microns by multiplying VDI by pixel size. VDI is the average vessel caliber and does not reflect the change in vessel density; therefore, VDI is sensitive to vascular dilation^154^.
Average and distribution of vessel tortuosity (VT)	1^a^	Skeletonized	Segment length along the centerline divided by endpoint linear distance. Can inform on pathological microvascular remodeling and/or ischemia^153^.
Branchpoint density (BD)	nodes/mm	Skeletonized	Number of identified branchpoints divided by total vessel length. This parameter describes the level of interconnection in the microvascular network and may indicate the resilience to occlusion or blockage of blood flow^153^.
Fractal dimension	1	Binarized	Calculated based on box method. Identifies the extent to which structures within the microvascular network repeat across a range of length scales^153^. Altered spatial distribution of the capillary network is an indicator of impairment of oxygenation and nutrient delivery^173^.
Non-flow area	mm^2^	Binarized	Total number of black pixels enclosed by the contour selected by the user as lacking vessels for avascular areas other than FAZ. Provides information on microvascular network defects resulting in focal decrease due to localized absence and/or collapse of retinal capillaries^150^.
Foveal avascular zone (FAZ) area	mm^2^	Binarized	Total number of black pixels enclosed by the FAZ segmentation contour.
FAZ perimeter length	mm	Binarized	Length of the perimeter of the FAZ.
FAZ axis ratio	1	Binarized	Ratio between the major and minor axis of the ellipse best fit to FAZ outline shape.
Acircularity index	%	Binarized	Ratio between the measured perimeter and the perimeter of a circular area of the same size.
Foveal vessel density 300 (FD-300)	%	Binarized	Percentage of the area occupied by vessels (white pixels) divided by the area in a 300-µm width rim surrounding the FAZ. The rim width value was chosen based on the relationship between FAZ and ganglion cell complex thickness in normal eyes to better distinguish between normal variations in FAZ and those due to pathology.
Flow area	mm^2^	Binarized	Ratio of the area occupied by vessels (white pixels) divided by a region of interest chosen by the user. Used to quantify the area of flow signal within a user-predefined outer retina (vessel-free region between the outer plexiform layer and Bruch’s membrane).

^a^Unit of 1 indicates dimensionless.

Currently, the quantitative analysis of the retinal microvascular network in OCTA is limited to the utilization of 2D-projection maps. This approach facilitates direct comparison with other 2D techniques, such as FA and ICGA, and compresses projection artifacts discussed under the subsection on Image Artifacts above. The most-used metrics are vessel density, flow area, non-flow area and FAZ area. However, the way in which such metrics are defined and used can vary between instruments and/or studies.

Munk et al. have reported a plethora of terminology around reduced flow. Altered retinal and choroidal capillary flow/perfusion in diabetic retinopathy has been described as “flow void”, “capillary non-perfusion”, or “impaired capillary perfusion”^5^. Other terms used include “greyish area”, “no-flow area”, “areas with decreased vascular perfusion”, “flow deficit”, “flow attenuation”, “low and no flow”, “reduced flow”, and “flow abnormalities”^5^. Similarly, Fawzi reported that a whole range of terms have been used to describe the absence of flow, including “nonperfusion”, “avascular area”, and “absent flow”^17^. There are further inconsistencies among metrics, with definitions of vessel density referring to areas or lengths. The “vessel density” quantified by built-in software of the Zeiss Angioplex OCTA instrument is defined as the total length of skeletonized perfused vasculature per unit area of the measurement region. The Optovue AngioVue OCTA built-in software calculates vessel density as the total area of perfused vessels (“all white pixels”) per image area. Durbin et al. defined “perfusion density” as the percentage area occupied by perfused binarized vessels and “vessel density” based on a map with vessels of 1-pixel width^155^.

After consideration of these and other works, we recommend using the metrics as named and defined in Table 1 and visualized in Fig. 8. Our selections appear most commonly in the literature and comprehensively characterize the important changes in the vascular network architecture due to vascular dysfunction^153^. Specifically, we suggest replacing the term vessel density with vessel area density (VAD) to better distinguish between VAD and vessel length density (VLD) (Table 1).

The FAZ plays an important role in microvascular disease development and should be quantified along with the microvascular network. Most commercial instruments and published papers report FAZ area using the same definition. However, in some studies, FAZ area is analyzed within a single retinal layer; whereas, in others from the whole retina. Since such differences will impact on the analysis outcomes, comparisons between such studies would be invalid. We recommend adding other FAZ metrics, including FAZ perimeter length and acircularity index^156^. The strong correlation between FAZ acircularity and the presence of diabetic retinopathy has been demonstrated by Krawitz et al.^156^. The parameter “foveal vessel density (FD-300)” should be also considered, first introduced by Wang et al.^157^ and adopted by Optovue. The parameter is defined as the percentage of the area occupied by vessels (white pixels) divided by the area in a 300-µm width rim surrounding the FAZ and was reported to be more sensitive to FAZ enlargement introduced by vascular pathologies than FAZ area alone^157^. A similar observation was made by Rosen et al., who have performed a similar analysis to Wang et al. but using a 200-µm width rim^158^.

We recommend at this stage in the development of OCTA using the widest applicable range of standardized OCTA metrics. The comprehensive characterization of the retinal microvascular network will provide the strongest prospects for identifying the best biomarkers for early diagnosis and monitoring of diseases. As yet, which metrics have the highest predictive value in clinical practice is unknown^159^; therefore, this approach will support the identification of the best metrics and, ultimately, the best diagnostic tools, including those based on artificial inteligence^111^. Further systematic research on biomarker refinement will eventually lead to identifying single or minimum combination biomarkers to precisely identify disease stages and response to treatment.

For consistency, the definitions of the retinal layers proposed above should be used along with these metrics to clearly identify which part of the vascular network is being characterized. The retinal microvascular network architecture should be characterized in the whole image as well as in subfields defined by the Early Treatment Diabetic Retinopathy Study (ETDRS) grid commonly applied in ophthalmology. This grid divides the retina into nine regions defined by three rings: a central foveal ring 1 mm in diameter, an inner macular ring 3 mm in diameter, and an outer macular ring 6 mm in diameter. The inner and outer rings are divided into four quadrants: nasal, temporal, superior, and inferior^160^. An advantage of the ETDRS grid is that its layout accounts for natural differences in the shape of the retina^160^; enables localization of important areas of the macula and identifies their changes due to disease; and is applied by many ophthalmic imaging modalities; thus, allowing direct comparison of localized changes between OCTA and other modalities.

## Clinical practice

Standardization is needed across the whole OCTA measurement pipeline, thus, we recommend adoption of a standard protocol for imaging subjects: pupil dilation before OCTA examination (after careful assessment by the treating physician for potential harmful side effects) and lubricating eye drops (for subjects with an unstable tear film); imaging in a dark room with OCTA imaging head adjusted to ensure subject comfort; subject fixation on a central target and requested to refrain, where possible, from blinking; and imaging conducted by well-trained OCTA operators. OCTA images should be reviewed immediately post-measurement and repeated if determined to be inadequate. We recommend the combination of visual inspection (presence of blinking and motion artifacts) and an image quality indicator (scan quality index or equivalent) to confirm poor image quality or otherwise. Subjects attending single clinics should be imaged on the same device with the same protocols to increase the accuracy of comparison between visits^63^. Viewing en face OCTA maps should always be performed in concert with inspection of 3D and single B-scan OCT quality and segmentation results to ensure correct image interpretation^10,38,63^. Ideally, OCTA instrument vendors would harmonize training to follow standardized approaches to data collection, analysis and interpretation.

In the meantime, clinicians and researchers must keep in mind that their data might not be directly comparable: between institutions, instruments, and their own datasets analyzed using different manufacturers’ software. Currently reported VAD in the superficial vascular complex can range from 28 to 60% between studies^49^. Yu et al., using perfusion-labeled human donor eyes (ex vivo), have reported average superficial vascular complex VAD of circa 31%^161^, suggesting that lower numbers might be closer to true values. Sampson et al. have demonstrated that a recent update of the RTVue RX AngioVue software has resulted in a systematic reduction in the measured VAD^162^. In general, there needs to be more open discussion of differences in quantitative metrics and their origins, and we repeat that more effort should be undertaken to harmonize imaging protocols and data analysis methods to minimize these differences.

When reporting intrasession (imaging on the same day) and intersession (imaging on different days) repeatability, we recommend measuring the coefficient of repeatability, *CR*, based on the within-subject standard deviation, *S*_*w*_, as introduced by Bland and Altman^88^. Here, the standard deviation of repeated measures for each subject should be calculated and squared to obtain the variance for each subject. *S*_*w*_ is the square root of the average variance for all subjects. *CR* = 2.77·*S*_*w*_ and 95% confidence intervals (CI) can be calculated as: $CR \pm 1.96 \cdot \left( {S_w/\sqrt {2n(m - 1)} } \right)$, where *n* is the number of subjects and *m* the number of measurements for each subject. Furthermore, the inter-ocular symmetry (differences in metrics between the left and right eye) can be quantified by limits of agreement, *LA*, also introduced by Bland and Altman. Here, the mean, $\bar d$, and standard deviation, *SD*, of the differences between both eyes is first calculated to then be able to calculate *LA* as: $\bar d \pm 1.96SD$. Some authors do not report the within-subject standard deviation necessary for estimating the threshold of test-retest variability^163^. Others define *CR* as 1.96 times the standard deviation of the differences, instead of 2.77 times the within-subject standard deviation, as recommended by Bland and Altman^164^. Such varied approaches to statistical analysis of results preclude meaningful comparisons between studies^88^.

## Recommendations, future perspectives, and challenges

OCTA has greatly advanced in recent years, yet the journey toward clinical utility is just beginning. The prospects for OCTA and its applications are numerous and exciting if we can move beyond fragmented and piecemeal approaches toward large-scale coordinated application of a consensus methodology.

An exciting prospect is the building of reliable, multi-sourced retinal OCTA databases of healthy subjects and patients with key vascular diseases^165^. This work has commenced with Coscas et al., who first reported a single-center normative database of OCTA metrics^166^. Still more comprehensive databases are required not only to further the OCTA-based diagnosis of retinal disease but also support the discovery of better biomarkers with artificial intelligence and related technologies^25,167^. To be able to build reliable large-scale databases^58,168^, we have outlined changes in practice and the further research required. We summarize the most important recommendations of this review below.

1. *Consistent anatomy*: Adopt definitions and terminology for the retinal vascular layers as summarized in the section “Retinal layer segmentation and definition” above. Such consistency will minimize variations in data analysis and reporting of findings due to variable layer definition and in-depth location of en face OCTA maps and make easier comparison of findings between reports.
2. *Report key imaging parameters*: Producers of OCTA instruments and researchers should report the transverse resolution and transverse scanning density along with the angular FOV of each protocol. As well, the range of accessible vessel velocities (or interscan time) should be reported. Only by knowing the full set of parameters, can OCTA users fully understand the limitations of the instrument applied and properly interpret their results.
3. *Define image quality metric*: Further work should be undertaken to define the best-automated method for assessment of OCTA image quality recommended to be followed by all.
4. *Standardize magnification correction*: Further investigation should be undertaken to arrive at the most practical method for correction of OCTA image transverse magnification to ensure universal uptake by all users. The established method should be implemented in commercial and non-commercial OCTA instruments.
5. *Correct projection artifacts for deep vascular complex*: To allow the accurate assessment of the deep vascular complex, algorithms that enable the removal of projection artifacts should be first optimized and then adopted.
6. *Standardize OCTA image processing and segmentation*: Systematic studies should be undertaken to test and validate algorithms for automated OCTA image processing: en face image thresholding and vessel segmentation; and segmentation of the retinal layers. Such studies should be applied to data from all types of OCTA instruments with algorithm performance reported by efficiency, accuracy, and execution time^89^. An optimal, single pipeline for OCTA image processing and segmentation will vastly improve the starting point for assessment of quantitative metrics.
7. *Standardize image averaging*: Systematic studies should be undertaken to evaluate the importance of en face OCTA image averaging on the accuracy of OCTA vascular metrics. Based on the outcomes, a standard imaging protocol should be adopted to minimize variations in metrics caused by averaging.
8. *Create more open access data sets*: More open access data sets are required to enable validation of protocols. Currently, there are two such OCTA datasets available, from the ROSE^89^ and PREVENT^105^ studies. Such data sets should include data collected with OCTA but ideally also with histology; histology currently suffers from less artifacts and enables visualization of many more vessels^68^. We acknowledge that patient privacy concerns might stop researchers from building and sharing large databases between institutions^169^. However, large data sets are critical for the data-driven innovation that ultimately will improve patient care^169,170^. New tools and better regulations/policies need to be developed to better protect privacy and enable data sharing^169^. Possible tools for considerations are describing patterns of groups in the dataset rather than individuals, federated learning^171^—a machine learning paradigm in which algorithms are trained collaboratively without exchange of data—as well as generative adversarial networks^169,172^—a deep learning approach in which a model is trained to generate synthetic image examples that are different from the original images.
9. *Create open-source software and standard metrics*: Following Steps 1–8 above should lead to a suite of best practices suitable for implementation in open-source software that will also enable quantitative analysis of the retinal vascular network—based on a selection of the metrics defined herein thereby minimizing errors of using and comparing data analyzed by different algorithms^97,99^.

In the meantime, OCTA users should focus their efforts on harmonizing imaging protocols, data analysis methods, and reporting of data and in ensuring they publish detailed descriptions of all procedures they apply.

We now move our attention to motivating continued research on promising new ways of quantifying the microvascular network architecture.

Visualization of retinal OCTA data through a 2D-projection map is convenient as it enables direct comparison with other 2D techniques, such as FA and ICGA, and compresses projection artifacts as discussed. However, 2D-projection of the volumetric vascular network may lead to altered metrics, such as caused by false branch points generated by vessels appearing to overlap in 2D but in reality existing at distinct elevations in 3D, and introducing errors into other metrics such as segment length^153^. 3D vessel orientation has been reported to be important in understanding local tissue oxygenation^153,173^. In principle, full volumetric analysis would seem a laudable objective, but much more work is needed to advance methods and metrics for characterization of the retinal microvascular network in 3D before it could be seen as a viable clinical pathway^174,175^.

Further research on increasing the visibility of retinal vessels in the OCTA image is warranted. Imaging artifacts, image post-processing, and vessel orientation to the scanning beam all impact on the visualization of retinal vasculature^86,176^. As demonstrated by Zhu et al.^176^ and further confirmed by Yu et al.^68^, vertically oriented (i.e., aligned with the optical beam) vessels are more difficult to visualize. Possible ways to improve visualization include: imaging after tracer injection, e.g., intralipid^176^; image averaging^177^; multi-angle illumination and detection^178^; and expanding the detectable flow velocity range by imaging at various scanning rates and combining images obtained at different rates^50,179–181^. Varying dynamic and static wavefront aberration may introduce variable image quality in OCTA images as well as artifacts, such as double rendering of vasculature, that could be overcome by applying adaptive optics correction^66^.

A range of other technical improvements merit further research. Increasing the angular FOV to more than 100 degrees, without sacrificing sampling density, would enable monitoring and assessment of vascular conditions at different eccentricities^182^. A recent study has shown that the early stage of diabetic retinopathy occurs in the peripheral retina so can be missed by standard FOVs^24^.

A dual-mode instrument incorporating standard (20 µm) and high (5–10 µm) transverse resolution would allow users to choose between screening and region-of-interest imaging. A promising pathway to a dual-mode instrument is to combine OCTA with adaptive optics to reduce the impact of the optical aberrations of the human eye^53,183–185^. More generally, higher-scan-speed and higher-resolution OCTA imaging, if artifacts could be overcome, would further improve our capacity for functional study of the retinal microvasculature^49^ and further enhance early-stage diagnosis of retinal vascular diseases^186,187^.

Whilst we have promoted a wide range of metrics as potential biomarkers, we should not preclude future development of new metrics. In this vein, Ma et al. have recently proposed the retinal microvascular orientation pattern as a potential biomarker for diabetic retinopathy diagnosis^188^.

## Conclusion

Our review highlights the challenges regarding the lack of comparability of OCTA findings between instruments and studies and the importance of drastically improving this to support the diagnosis, monitoring, and treatment of retinal and systemic vascular diseases. The OCTA retinal imaging community is urged to define best practices for data collection, analysis, and reporting of results, and to promote transparent data sharing to promote accuracy, reliability, and wider collaboration. We hope that the topics discussed here, in proposing minimum standards, as well as identifying areas of concern and opportunity for future research, will encourage researchers, clinicians, and systems manufacturers to come together as a community to continue harmonization of the current disparate terminology, methods, and practice and motivate progress in the field.

OCTA is a powerful and exciting technology that has the strong potential to change medical practice in relation to retinal disease. We second Spaide et al. who wrote: *“OCTA development can and will be a group effort. Let’s get to work”*^10^*.* Indeed, together, we can do it.
